# Competitive assembly of South Pacific invasive ant communities

**DOI:** 10.1186/1472-6785-9-3

**Published:** 2009-01-24

**Authors:** Philip J Lester, Kirsti L Abbott, Megan Sarty, KC Burns

**Affiliations:** 1 School of Biological Sciences, Victoria University of Wellington, P.O. Box 600, Wellington, New Zealand; 2 Science Faculty, Monash University, Melbourne, VIC, Australia; 3 MAF Biosecurity New Zealand, P.O. Box 2526, Wellington, New Zealand

## Abstract

**Background:**

The relative importance of chance and determinism in structuring ecological communities has been debated for nearly a century. Evidence for determinism or assembly rules is often evaluated with null models that randomize the occurrence of species in particular locales. However, analyses of the presence or absence of species ignores the potential influence of species abundances, which have long been considered of major importance on community structure. Here, we test for community assembly rules in ant communities on small islands of the Tokelau archipelago using both presence-absence and abundance data. We conducted three sets of analyses on two spatial scales using three years of sampling data from 39 plots on 11 islands.

**Results:**

First, traditional null model tests showed support for negative species co-occurrence patterns among plots within islands, but not among islands. A plausible explanation for this result is that analyses at larger spatial scales merge heterogeneous habitats that have considerable effects on species occurrences. Second, analyses of ant abundances showed that samples with high ant abundances had fewer species than expected by chance, both within and among islands. One ant species, the invasive yellow crazy ant *Anoplolepis gracilipes*, appeared to have a particularly strong effect on community structure correlated with its abundance.  Third, abundances of most ant species were inversely correlated with the abundances of all other ants at both spatial scales. This result is consistent with competition theory, which predicts species distributions are affected by diffuse competition with suites of co-occurring species.

**Conclusion:**

Our results support a pluralistic explanation for ant species abundances and assembly. Both stochastic and deterministic processes interact to determine ant community assembly, though abundance patterns clearly drive the deterministic patterns in this community. These deterministic patterns were observed at two spatial scales. Results indicate that abundance-based null models may be more sensitive in detecting non-random patterns in community assembly than species co-occurrences analyses.

## Background

Opinions on the processes governing community assembly are polarized [1-3]. One school of thought maintains that ecological communities are assembled deterministically according to 'assembly rules', generated by biotic interactions [4-6]. An opposing view asserts that communities are assembled stochastically by chance dispersal of species with life history characteristics suited to local environmental conditions [7-9]. The relative importance of chance and determinism in structuring ecological communities has been fiercely debated for nearly a century and we are far from a resolution to the debate.

Diamond [5] hypothesized that interspecific interactions result in community assembly rules. He suggested that some pairs of species never coexist, either by themselves or as part of a larger combination. Evidence for assembly rules are often evaluated with null models that randomize the occurrence of species in particular locales. However, analyses of the presence or absence of species ignore the potential influence of species abundances, which have long been considered of major importance on community structure [10-13]. Abundance effects may be particularly important in determining the distribution of invasive species [14]. Less abundant invasive species likely have weaker ecological effects on community assembly than more abundant species. Nevertheless, the effects of species abundances have largely been ignored when testing for community assembly rules.

Patterns in community structure, and their underlying mechanisms, can differ among spatial scales [15], as can our understanding of the invasiveness of introduced species. For example, investigations over larger spatial scales often incorporate a variety of habitats, some of which may not be suitable for an invasive species and thus provide refuges for native species. Despite evidence that community assembly rules can be scale-dependent [16,17], null model tests for community assembly rules are typically assessed at single scales [18].

Here, we test for community assembly rules in ant communities on islands in the South Pacific, by conducting abundance-based null model analyses at two spatial scales. Ant communities on Islands in the South Pacific are often composed of introduced species, which are an ideal group for the study of processes governing community assembly. Interspecific aggression [19], chemical warfare [20], dominance hierarchies and competitive displacement [21-23] have been documented in numerous ant species inhabiting different parts of the globe. In addition, many invasive ants are considered superior competitors [24]. Most islands in the South Pacific are volcanic in origin, so their flora and fauna are derived from over-water dispersal. Unlike most other island colonists, most ant species have colonized islands comparatively recently, with the aide of human travellers, and the process of island colonization and ant community assembly continues [25]. Because South Pacific ant communities are often comprised almost entirely of introduced species, they provide an ideal opportunity to study how ecological communities are assembled. Unfortunately, they might also provide a template for the future of many other ecological communities, as invasive species continue their global spread.

We censused ant communities in 39 plots on 11 islands and used null models to test for community assembly rules on two spatial scales (i.e. within and among islands). First, we randomised the presence of ant species among samples to test for negative co-occurrence patterns. Second, we randomised the presence of individual ants among samples to test whether ant species richness was inversely related to total ant abundances. Third, we randomised the presence of individual ants among samples to test whether the abundances of each ant species was inversely related to the abundance of all other ant species.

## Methods

### Study site, species and field sampling

**Figure 1:**
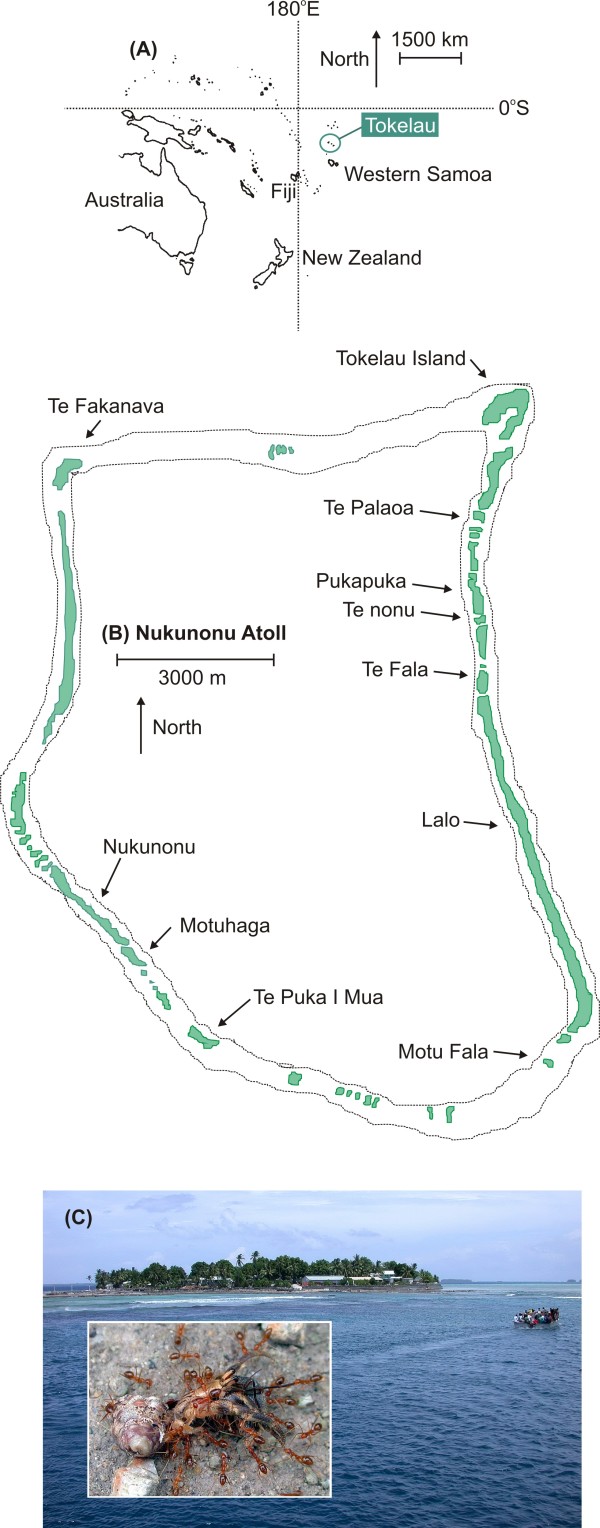
**Tokelau and the islands used in our study**. (A) A map of the South Pacific showing the relative location of Tokelau. (B) A map of Nukunonu Atoll showing the location of the 11 islands used in our study. (C) A photograph of Fakaofu Atoll of Tokelau. All three Tokelau Atolls are similar, with only one or two inhabited islands and many uninhabited islands such as in the background of this picture. The inset picture is of the yellow crazy ant (*Anoplolepis gracilipes*) consuming a dead hermit crab (*Coenobita *sp.).

Tokelau lies 483 km north of Samoa in the Pacific Ocean (approximately 9°45'S, 171°35'W) and is comprised of three low-lying coral atolls: Atafu, Nukunonu and Fakaofo. The atolls are 50–100 km apart and each is made up of 38–51 islands surrounding a shallow lagoon, one or two of which are permanently inhabited by people on each atoll (Fig. 1). The islands are generally small, with Tokelau's total land area being approximately 12 km^2^. Tokelau lies in the southeast trade wind belt and has a humid tropical climate that displays little seasonal variation (mean annual temperature 28°C, mean annual rainfall 3000 mm [26]). The islands of Tokelau are comprised of coral rubble of varying size with poorly developed soil overlying beach rock (Parham 1971). The islands are low-lying (~5 m above sea level) and narrow. The vegetation is low in diversity and typical of small Pacific atolls [26,27].

Ant communities on islands in the Southern Pacific Ocean typically have few native species and are often comprised entirely of species that were brought their by human travellers [25]. Only 28 ant species have been recorded on Tokelau and of those only two are Pacific endemics, with no species endemic to Tokelau [28].

Censuses were conducted during three successive visits to the archipelago (November 2002, November–December 2004 and June–July 2005). A standard sampling design was used throughout [29-31]. Survey plots consisted of 15 × 15 m quadrats located at least 40 m apart in forested areas. The number of quadrats per island ranged from between two and nine over the period of 2002–2005. Four to five pitfall traps were haphazardly situated throughout the plot and one-third filled with Gault's solution, which is an insect killing agent and preservative [32]. Placement of traps was haphazard (rather than random) so as to avoid traps being placed beside or on individual ant nests, which could provide biased estimates of ant abundances. The pitfall traps were plastic containers 9 cm tall, 7.5 cm diameter at the top, tapered to 5 cm diameter at the bottom and placed flush with the ground surface. Traps were only left out for 24 hrs due to the speed at which they occasionally accumulated abundant ant species, such as *Anoplolepis gracilipes*. In total we placed a total of 184 pitfall traps, within 39 plots from 11 islands. Analyses at the "plot" scale refer to the pooled number and abundance of each species from all pitfall traps within each quadrat. Analyses at the "island" scale refer to the pooled number and abundance of each species from all quadrats within each island, sampled over the period of 2002–2005.

### Species co-occurrences

To test for negative co-occurrence patterns, we used standard analyses in the freely available software package EcoSim [33]. In this analysis, there were 17 rows of species and either 11 or 39 columns for islands or plots, respectively. Negative co-occurrence patterns were quantified with three indices: the C-score index, the checkerboard score and the number of unique species combinations. The C-score is obtained by calculating the number of checkerboard units *cu *for each species pair. For example, at the island scale of our analyses: *cu *= (*o*_*i *_- *s*)(*o*_*j *_- *s*), where *o*_*i *_is the total number of islands occupied by species *i*, *o*_*j *_is the total number of islands occupied by species *j*, and *s *is the number of islands occupied by both species [34]. A single C-score, which describes community-level species co-occurrences, is obtained by averaging *cu *values of all species pairs. The checkerboard score is obtained by tallying the number of species pairs that never co-occur with one another. The number of unique species combinations is a count of all species pairs that co-occurred with one another.

To test for non-random co-occurrence patterns, the observed values of each index were compared to values generated in 5000 simulations of the observed species × plot presence-absence matrix using fixed row and column sums. The sequential swap technique was used to generate random permutations of the observed matrix. Miklós & Podani [35] show that this method biases against support for non-random co-occurrence patterns and provides a more conservative test of the co-occurrence assembly rule. If communities are structured deterministically according to assembly rules, observed communities should have higher C-scores, fewer unique species combinations, and more checkerboard species pairs than expected under the null model. Among-island and within-island analyses were conducted separately.

### Species richness

Competition theory predicts that as the total number of individuals in an area increases, competition for resources leads to the exclusion of competitively subordinate species [10]. We tested this prediction by evaluating whether areas with higher numbers of ants had lower numbers of ant species. To test for an inverse relationship between species richness and overall ant abundances, we began by obtaining expected values of species richness for each sample locale (i.e. plot and island). Expected values of species richness were obtained using rarefaction, which estimates species richness on a per-individual, rather than a per-area, basis [2,36,37]. To generate rarefaction curves we used the programme RAREFACT 1.0 [38]. Different numbers of ants were randomly sampled from the total pool encountered during sampling using this computer simulation, which estimates species richness for a given number of individual ants sampled. Separate rarefaction curves were generated for each spatial scale. The total pool of ants sampled across the archipelago was used to calculate expected species richness values for each island and the total pool found on each island was used to calculate expected species richness values for each plot.

This application of rarefaction yields expected values of species richness if individuals were randomly distributed among samples. Differences between observed (*O*) and expected (*E*) species richness values (*O *- *E*) therefore indicate whether samples sites have higher or lower richness than expected by chance. If ants compete for resources, sites with greater total ant abundances will contain fewer species than expected, resulting in negative *O *- *E *values. Conversely, sites with lower overall ant abundances will contain more species than expected, resulting in positive *O *- *E *values. However, differences between observed and expected values increase passively with the magnitude of expected values. To remove this confounding effect we divided the difference by expected values [(*O*-*E*)*E*^-1^], which results in an unbiased estimates of deviations from expected species richness values.

General linear models were used to establish whether differences from expected species richness varied with total ant abundances. Standardised differences from expected species richness values were included in the model as a dependent variable. Total ant abundances were treated as a covariate. Separate analyses were conducted for each scale (i.e. plot or island). In the plot-scale analysis, island was included in the model as a random factor to account for the independence problem generated by multiple plots occurring on the same island. Data were log_10 _transformed to conform to normality and homoscedasticity assumptions at the island-scale. Data conformed to assumptions without transformation at the plot-scale. Analyses were conducted in SPSS [39].

### Abundances

We generalized linear models to test whether the abundance of each ant species declines with the abundances of all other ant species. Observed abundances of each species were used as the dependent variable. The sum of all other ant species present in each sample (i.e. plots or islands) was treated as a covariate. The expected abundance of each species in each sample was treated as a second covariate (*E*_*ij*_). To calculate the expected abundance (*E*) of each species (*i*) in each sample (*j*), we multiplied the total abundance (*A*) of that species ($\sum_{j=1}^{n}A_{i}$) by the abundance of all ant species in that sample ($\sum_{i=1}^{n}A_{j}$). This product was then divided by the total number of all ants found at that scale [($\sum_{i=1}^{n}\sum_{j=1}^{n}A_{ij}$)] to obtain:

$$E_{ij}=\frac{\left(\sum_{j=1}^{n}A_{i})(\sum_{i=1}^{n}A_{j}\right)}{\sum_{i=1}^{n}\sum_{j=1}^{n}A_{ij}}$$

A significant effect of expected ant abundances would suggest stochastic processes shape community assembly. Support for community assembly rules would require negative relationships between the observed abundance of each species and the summed abundance of all other ant species. Ant species was included as a random factor in the island-scale analysis to account for the independence problem associated with including more than one value for each species. Both ant species and island were included as random factors in the plot-scale analysis to account for the independence problem associated with including more than one value for each species and each island. Separate analyses were conducted at each scale using the generalized linear model procedure with a Poisson distribution in SPSS [39].

## Results

Seventeen species were found over the three years of sampling (Table 1). Total ant abundances varied over three orders of magnitude (5–5,000 ants) among plots. One ant species (*Anoplolepis gracilipes*) was particularly abundant. It accounted for an average of 50% of all ants sampled among islands and variation in total ant abundance was associated with *A. gracilipes *abundance. The total number of ants observed was highly correlated with the total number of *A. gracilipes *at both the quadrat (Pearson's *r = *0.940;*P *< 0.001) and island scales (Pearson's *r = *0.914;*P *< 0.001).

**Table 1:** Ant species with abundances found in plots and islands.

Species	Plots		Islands	
		
	Presence	Abundance	Presence	Abundance
	(n= 39)	range	(n= 11)	range
**Subfamily Formicinae**				
*Anoplolepis gracilipes *(Smith)	69%	3–10541	75%	77–41296
*Paratrechina longicornis *(Latr.)	13%	4–52	25%	4–78
*Paratrechina vaga *(Forel)	31%	1–8	58%	1–41
				
**Subfamily Myrmicinae**				
*Cardiocondyla nuda *(Mayr)	5%	1–6	25%	1–7
*Monomorium floricola *(Jerdon)	31%	1–4	58%	1–19
*Monomorium liliuokalanii *Forel	3%	1	17%	1
*Pheidole fervens *Smith	31%	1–24	75%	1–75
*Pheidole oceanica *Mayr	56%	1–26	75%	2–156
*Pheidole sexspinosa *Mayr	36%	1–7	58%	1–38
*Pheidole umbonata *Mayr	41%	1–18	67%	1–88
*Rogeria stigmatica *Emery	13%	1	50%	1–5
*Strumigenys *sp. 1	5%	1	25%	1–2
*Tetramorium bicarinatum *(Nyl.)	18%	2–35	33%	6–103
*Tetramorium lanuginosum *Mayr	41%	1–139	58%	1–424
*Tetramorium simillimum *(Smith)	18%	1–35	42%	1–84
*Tetramorium tonganum *Mayr	15%	1–14	33%	2–32
				
**Subfamily Ponerinae**				
*Anochetus graeffei *Mayr	18%	1–5	50%	1–13

The 39 plots utilized in the study were placed on the 11 islands examined from Nukunonu Atoll, Tokelau.

### Species co-occurrences

Patterns of species co-occurrences were scale-dependent. No evidence for co-occurrence assembly rules were found at the analysis scale of among islands. The observed C-score (4.368) was not statistically different from null model expectations among islands (4.369, 0.006; mean, σ; *P *= 0.485). Similar results were obtained for the number of checkerboard species pairs (observed = 13, expected = 15.997, 9.985; *P *= 0.882) and the number of unique species combinations (observed = 11, expected = 10.999, 0.028; *P *= 0.998). In contrast, within-island analyses showed evidence for co-occurrence assembly rules. The observed c-score (41.007) was significantly greater than null model expectations (40.214, 0.151; *P*= 0.023). While the observed number of checkerboard species pairs (32) did not significantly differ from expected numbers (34.099, 11.918; *P *= 0.760), the observed number of unique species combinations (35) was lower than null model expectations (39.623, 0.376; *P *< 0.001).

### Species richness

**Figure 2:**
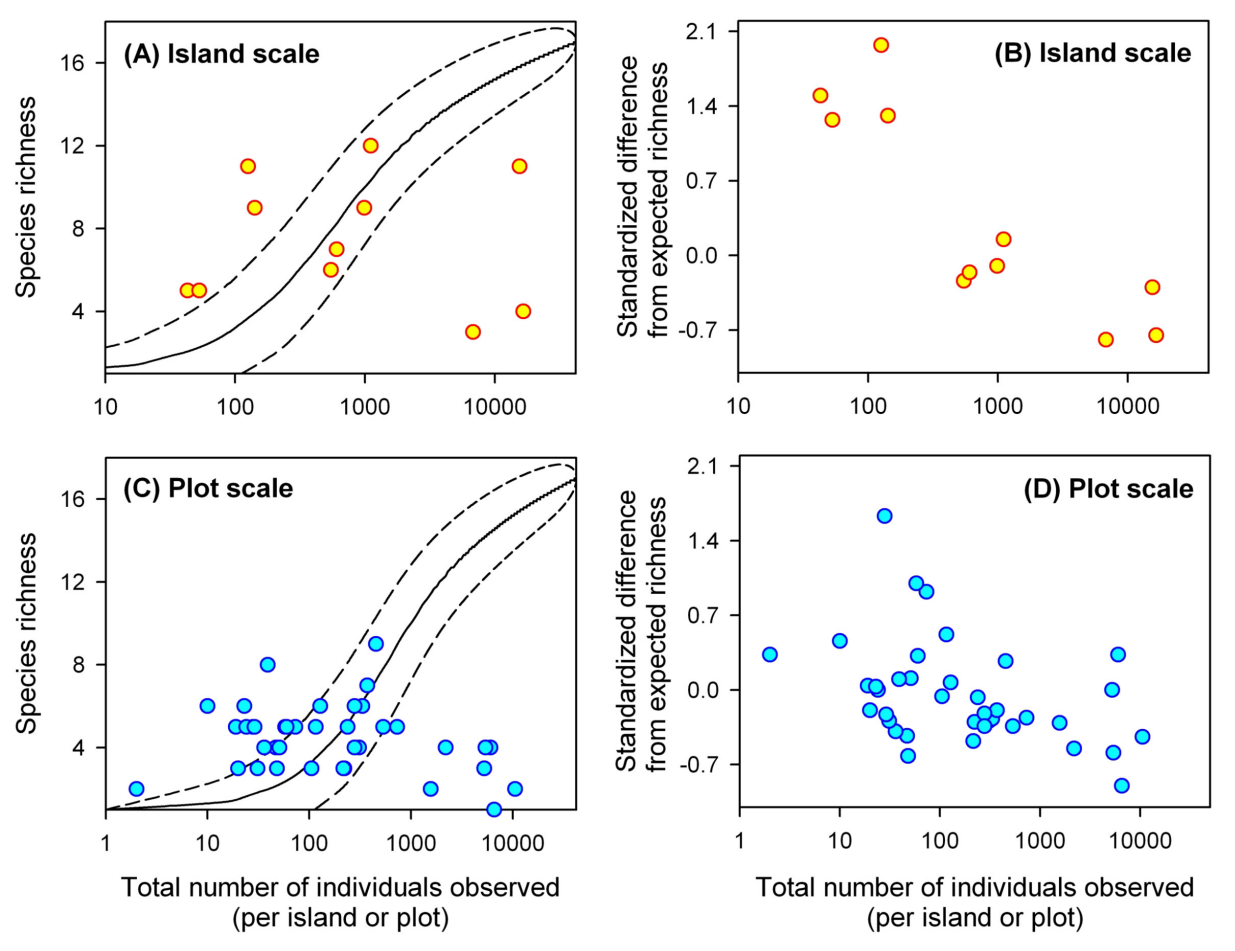
**Patterns in species richness of the Tokelau ant fauna**. On the left, total species richness is plotted against the number of individuals sampled. Each point represents a single sampling locale. Solid lines are rarefaction curves reflecting relationships between cumulative species richness and the number of individuals encountered among samples and dashed lines are 95% confidence intervals. On the right, deviations from expected species richness vales [(*O*-*E*)*E*^-1^] are plotted against total ant densities. Among island analyses are shown on top (*N *= 11). Among plot analyses are shown on bottom (*N *= 39). The rarefaction curve for the island scale is shown for both island and plot graphs for aesthetic purposes, rather than calculating many individual, plot scale, rarefaction curves.

Observed ant species richness deviated markedly from expected richness values generated by rarefaction analyses at both scales. Differences between observed and expected species richness values were associated with total ant abundances. At the among-island scale, deviations from expected species richness values were negatively correlated with total ant abundances (F_1,9 _= 31.660, *P *< 0.001) (Figs. 2A &2B). At the within-island scale, deviations from expected values of species richness were again negatively correlated with total ant abundances (F_1,27 _= 13.330, *P *= 0.001) (Figs. 2C &2D). Therefore, samples (i.e. plots and islands) with large numbers of ants had fewer numbers of species than expected under the null model.

### Abundances

**Figure 3:**
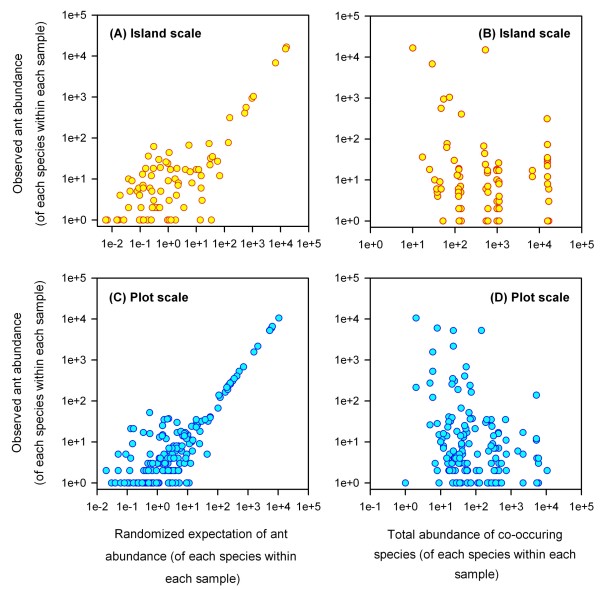
**Patterns of ant abundances in the Tokelau archipelago**. The abundance of each species within each sample is plotted against null model expectations (left) and the total number of co-occurring ants (right). Among island analyses are shown on top (*N *= 11). Among plot analyses are shown on bottom (*N *= 39). On both spatial scales ant abundances covary with randomized distributions, and are also negatively associated with the abundance of all other species.

Ant abundances were positively associated with null model expectations and negatively associated with the abundances of co-occurring species on both spatial scales. Analysis of ant abundances among islands showed that after accounting for species (Wald *X*^2 ^= 11,925, df = 16, *P *< 0.001), abundances increased with expected values (Wald *X*^2 ^= 52,802, df = 1, *P *< 0.001) (Fig. 3A) and decreased with the summed abundances of all other co-occurring species (Wald *X*^2 ^= 342, df = 1, *P *< 0.001) (Fig. 3B). Similar results were found in within-island analysis. After accounting for both species (Wald *X*^2 ^= 15,172, df = 16, *P *< 0.001) and island (Wald *X*^2 ^= 9,516, df = 10, *P *< 0.001), abundances increased with expected abundance values (Wald *X*^2 ^= 22,177, df = 1, *P *< 0.001) (Fig. 3C) and decreased with the summed abundances of all other co-occurring species (Wald *X*^2 ^= 96, df = 1, *P *< 0.001) (Fig. 3D).

## Discussion

The ant communities in the Tokelau Archipelago appear to be assembled deterministically. However, evidence for community assembly rules differed between abundance-based and community-based analyses. Species co-occurrences were distributed at random among islands, but evidence for negative co-occurrence patterns were observed within islands. The number of species present in samples at both spatial scales differed from randomized expectations; differences between observed and expected species richness values were negatively correlated with total ant abundances, indicating that areas with high ant abundances supported a reduced number of ant species. Population abundances of most ant species were correlated with both randomized expectations and the summed abundance of all other ant species. Therefore, consistent support for community assembly rules were found in abundance based analyses, but scale dependent results were found in analyses of species co-occurrences.

Co-occurrence analyses showed variable support for community assembly rules. Within islands, species tended to co-occur less frequently than expected by chance. Negative co-occurrence patterns are common in community ecology [40] and are commonly attributed to inter-specific competition. However, our results differ substantially from other analyses involving other invasive ants. Both red imported fire ants (*Solenopsis invicta*) and Argentine ants (*Linepithema humile*) have caused the disassembly of ant communities in other locales [17,41]. Our study involved several invasive species including *A. gracilipes*, which is thought to have similar effects on ant communities as *S. invicta *and *L. humile *[24]. Conversely, between island analyses indicated that species co-occurrences were distributed at random. This result is consistent with several other studies which found that community assembly rules can be scale-dependent [16,42]. Spatial scale thus appears to have a strong influence on the outcome of co-occurrence patterns. While there may be any number of hypotheses for this result, a plausible explanation is that analyses at larger spatial scales merge heterogeneous habitats that have considerable effects on species occurrences. Different species may have different habitat requirements. Determining the appropriate scale for co-occurrence analysis is probably community and habitat specific, and represents a difficult task.

Species richness analyses showed that the number of species present in samples declined with the total number of ants present. This result is consistent with theoretical predictions concerning how groups of competing species interact. Competition from groups of similar species may increase local extinction rates and decrease local colonization rates [5,43]. Islands and plots with large numbers of ants housed fewer species than expected by chance, whereas samples with smaller numbers of ants housed more species. Although several previous studies have documented similar patterns in island communities (see Burns [18]; and references therein) null model analyses that attempt to link community level patterns of abundance to species are rare. Therefore, the generality of the abundance-species richness relationship observed here remains unclear.

Rarefaction can overestimate species richness when spatial distributions are clumped [44]. If species interactions (e.g., competition) cause the spatial segregation of species distributions, the number of species present in samples should be less than predicted by rarefaction. We found that deviations from expected richness were negatively related to total ant abundances, suggesting that densely populated areas are subject to a greater influence of deterministic processes.

Patterns of the abundance of most ant species were negatively associated with the abundances of all other species. This result is consistent with competition theory, which predicts species distributions are affected by diffuse competition with suites of co-occurring species [5]. Patterns of ant abundances were also associated with null model expectations, indicating a stochastic component to ant community assembly. Random dispersal events and/or stochastic population dynamics may therefore help to determine ant abundance patterns, supporting recent work highlighting the effects of 'neutral' processes on spatial patterns of biodiversity [9]. When both relationships are viewed jointly, overall results support a pluralistic explanation for ant population abundances. Under this view, both stochastic and deterministic processes interact to determine ant community assembly. A similar conclusion has been reached by others examining ants on islands (e.g. Cole [19]), though a novel feature of our results is how abundance clearly drives deterministic patterns in this community.

Why patterns indicative of deterministic processes were consistently observed in abundance-based analyses, yet inconsistently in analyses of species co-occurrences is unclear. Perhaps the qualitative nature of binary data was not sensitive enough to detect evidence for assembly rules at all scales. Suites of analyses across a variety of scales using both quantitative and qualitative data may provide more accurate tests for assembly rules.

One ant species, the yellow crazy ant *Anoplolepis gracilipes*, appeared to have a particularly strong effect on community structure. When present in low abundances it appeared to have little to no effect on ant communities. However, in high abundance, *A. gracilipes *was associated with reductions in the number of co-occurring species and their abundance. Similarly, on Christmas Island *A. gracilipes *has its strongest influence on the communities when in high densities [45]. High abundances may help invasive ants such as *A. gracilipes *obtain resources through numerical superiority, but are also associated with increased aggression [46]. Our experimental work on these islands has demonstrated that when in high abundance, *A. gracilipes *often dominates preferred resources and only those species with different foraging modes or food preference can co-occur with them [31,47]. Spatial variation in the distribution of such dominant species also likely creates room for subordinate species, and the establishment of these subordinate species may have a large stochastic element [48]. Whatever the mechanisms for co-occurrence or exclusion, our results are consistent with competition theory, which predicts that interspecific competition and community structuring is abundance-dependent.

## Conclusion

Overall results showed that ant communities in the Tokelau archipelago are assembled deterministically. Although some support for stochastic processes was observed, most of our results are consistent with the hypothesis that competition structures community assembly, both within and among islands. Because South Pacific ant communities are often comprised entirely of introduced species, they may provide a template for the future, given the continuing global spread of invasive species.

## Competing interests

The authors declare that there are no competing interests.

## Authors' contributions

PJL designed the study, took part in the sampling, analyzed the data and drafted the manuscript. KLA and MS did the majority of sampling, helped analyze the data and write the manuscript. KCB lead aspects of the data analysis, helped develop the theoretical background and assisted in drafting the manuscript. All authors read and approved the final manuscript.
